# Spinal Epidural Hematoma and Gnathostomiasis

**DOI:** 10.4269/ajtmh.14-0579

**Published:** 2015-04-01

**Authors:** Verajit Chotmongkol, Amnat Kitkhuandee, Kittisak Sawanyawisuth

**Affiliations:** Department of Medicine and Surgery, Faculty of Medicine, Khon Kaen University, Khon Kaen, Thailand; Research Center in Back, Neck, Other Joint Pain and Human Performance (BNOJPH), Khon Kaen University, Khon Kaen, Thailand

**Figure 1.**
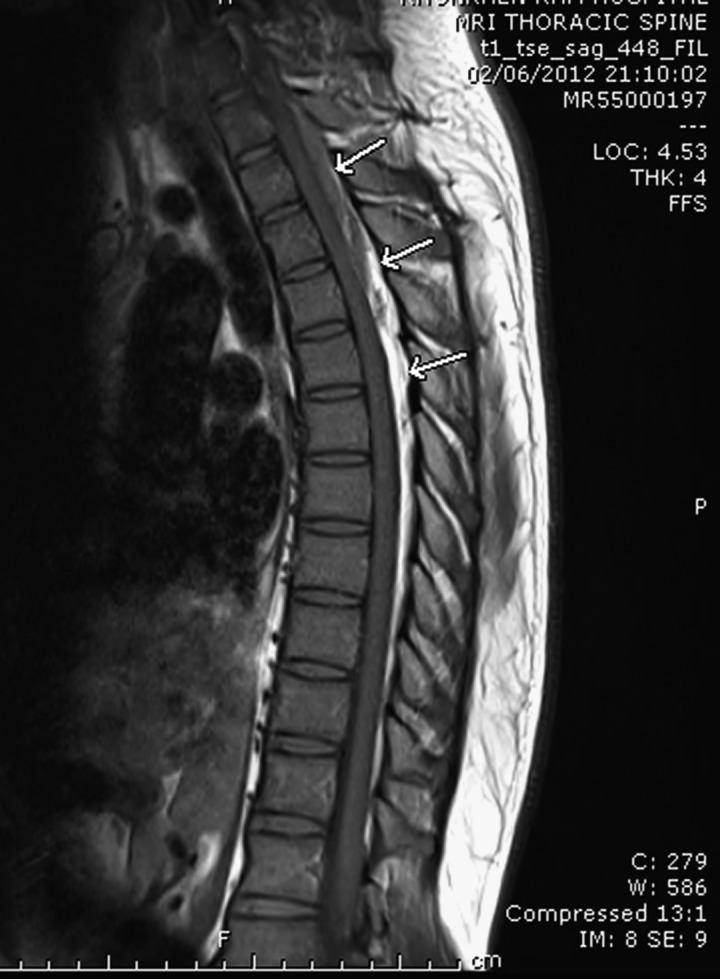
MRI of thoracic spine showed an isointensity lesion on the T1 weighted image at the posterior aspect of the thoracic thecal sac.

**Figure 2.**
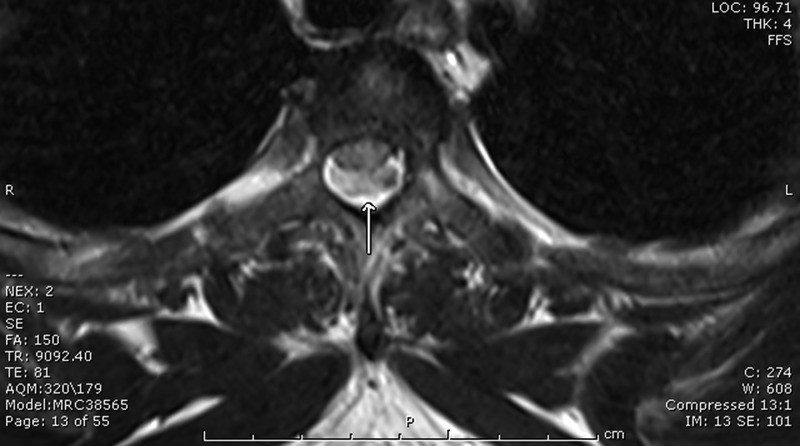
MRI of thoracic spine showed a mixed hyper- and hypo-intensity lesion on the T2 weighted image at the left posterior aspect of the thoracic thecal sac. The lesion compressed the thoracic spinal cord.

Typically, spinal gnathostomiasis is presented as radiculomyelitis.^1^ Here, we report a spinal epidural hematoma caused by *Gnathostoma spinigerum*. A 28-year-old man presented with acute chest pain for 1 hour. The pain was very severe, with dull aching on his left chest and toward his back. At the hospital, he developed paraplegia and sensory loss up to the T4 level. Physical examination revealed loss of proprioceptive sensation in both feet with loose rectal sphincter tone. Laboratory tests showed no peripheral eosinophilia, normal echocardiogram, and normal computed tomography (CT) angiogram of the aorta. Magnetic resonance imaging (MRI) of thoracic spine showed an isointensity lesion on the T1 weighted image (Figure 1) and mixed hyper- and hypointensity on the T2 weighted image at the left posterior aspect of the thoracic thecal sac (Figure 2). The lesion compressed to the thoracic spinal cord. The operative finding was epidural hematoma at the T4–T6 level. Blood serological study for gnathostomiasis using the immunoblotting technique was positive for the 24-kDa antigenic band.^2^ This diagnostic band has a sensitivity of 91.7% and a specificity of 100% compared with healthy control subjects.^2^ The patient received supportive treatment, and the weakness was gradually returned to normal within a few months.
